# An experimental and numerical study of the strength development of layered cemented tailings backfill

**DOI:** 10.1038/s41598-024-51464-2

**Published:** 2024-01-06

**Authors:** Nhleko Monique Chiloane, Fhatuwani Sengani, Francois Mulenga

**Affiliations:** 1 Department of Mining Engineering, University of South Africa, Florida Campus Private Bag X6, Johannesburg, 1710 South Africa; 2 Department of Geology and Mining, University of Limpopo, Private Bag X1106, Sovenga, 0727 South Africa

**Keywords:** Solid Earth sciences, Geology, Geophysics

## Abstract

The behaviour of a stratified backfilled stope in terms of strength development and stress distribution has not been well established in the field of rock engineering. Yet, the mining industries with massive ore bodies are looking into high production with a high standard of safety which is mainly governed by large excavation with backfill as a support system. It is difficult to fill these large excavations at one time. Therefore, a subsequent backfilling of the stope layer by layering is adopted, which results in a layered backfill structure. The purpose of this study was to explore the strength development, stress distribution and deformation across the stope supported by both layered and non-layered backfill. It has been observed that the backfill support system gain its strength with time, however, the layered backfill support system loses its strength when more layers are introduced, this is due to the shearing effect around the interfaces of the backfill layers. The impact of layering was validated by 3D numerical simulation. It is therefore concluded that non layered backfill support system are more suitable for stoping mining methods rather than layered support system.

## Introduction

The extraction of minerals in underground mines creates voids known as open stopes. These open stopes are filled with cemented tailings backfill (CTB) to prevent their failure, and thus, provide ground support to the surrounding rock mass^1–4^. CTB is made of mill tailings (70–85%), hydraulic binder (usually ordinary Portland cement) and mixing water (freshwater or mine-processed water)^5–7^. Due to these developments, several scholars have focused mainly on the strength design of CTB^8–10^^,^^11^. However, due to unique mining layouts, not all CTB designs will be one size fit all. For large stopes, it is difficult to fill the stope at one time. Therefore, a subsequent backfilling of the stope layer by layering is adopted^8,12–14^. Due to the structural differences between the complete CTB structure and the layered structure, the mechanical properties of both structures also differ. Therefore, the study of the strength properties of layered CTB would be greatly significant to characterize its mechanical properties.

The unconfined compressive strength (UCS) is usually the most preferred test to estimate the mechanical stability of CTB due to its simplicity, reliability and affordability^3,15^. Grice^16^ pointed out that CTB must have UCS of greater than 4 MPa when used as hangingwall support. In an attempt to improve the strength design of backfill support, several authors^8–10,17^ have undertaken extensive studies on different factors affecting the compressive strength of CTB. For example, Xiu et al.^17^ explored the effect of loading rate on the UCS behaviour of CTB. The group of authors employed five different loading rates, i.e. 0.1, 0.25, 0.5, 1 and 2 mm/min. Their findings reveal that the UCS of CTB samples increased respectively by 0.62%, 2%, 9.07%, and 16.62% as the loading rate was increased. The increase in UCS was attributed to the reduced time required for the material to respond to the strain. Also, the development of micro-cracks is restricted as the loading rate increases. Cao et al.^18^ support that the UCS of CTB increases with increasing loading rate. Similarly, Klein and Simon^19^ discovered that the addition of superplasticisers in CTB has a positive effect on the UCS of CTB. Haruna and Fall^20^ attest that CTB samples with superplasticisers demonstrate higher strength than samples without superplasticizers. Meanwhile, Fall et al.^21^ tested the strength of CTB samples cured under different temperatures. Their study shows that the UCS of CTB increases with increasing temperature regardless of the binder type. The high strength gain is attributed to the increased rate of formation of hydration products due to high temperature. Consequently, the hydration products consume excess water and reduce the porosity of the backfill. Thus, the strength of CTB increases^22,23^. Han et al.^24^ also add that the UCS of backfill increase with the curing period regardless of the binder content with the rise of temperature.

On the other hand, Liu et al.,^25^ developed a 3D model to analyse the stress distribution in a backfilled stope at different heights, namely, 15 m, 30 m, and 45 m. Based on the analysis of the study, the higher the stope, the higher the effective stress^25^. That is the stress within the backfilled stope increases with stope height. Whereas Nasir and Fall^26^ simulated the effect of stope on the strength development of CTB. The authors postulate that the strength development of CTB increases with stope size, particularly in the lower and middle parts of the stope. They further add that increasing the stope size leads to additional heat production from hydration, which enhances the strength of CTB. It is worth noting that these findings are based on the effect of heat due to cement hydration and heat transfer by considering stope size. Other worth mentioning authors^14,27,28^ have analysed the mechanical properties and failure pattern of layered CTB. Their fruitful studies show that the strength of CTB is significantly affected by the layering of CTB. That is, the strength of CTB was reported to reduce with increasing layers of backfill. Besides the mechanical properties of layered backfill, Gao et al.^29^ also looked at the shear characteristics of inclined layered backfill. Their study not only confirms that layering affects the integrity of CTB but also that as the layering angle increases from 20° to 25°, the shear strength and cohesion decreases by 35% and 43%, respectively. When it comes to energy dissipation during blasting of secondary stope, Sun et al.^30^ highlighted that as the number of layers increase, the absorption energy of the CTB increased gradually. In addition to these findings, the authors reported that the deformation of layered backfill mainly occurred at the layered position with a severe degree of fragmentation. In terms of failure modes, Chen et al.^31^ highlighted that the deformation mechanism of the layered surfaces in the UCS testing process was mainly the presence of higher monti-form bulges and the formation of new contacts.

In light of the previous studies documented above, questions may arise at this juncture. These pertain to the distribution of stress within a layered backfill stope, the impact of layering on the strength and performance of the surrounding rock mass throughout the stope, and the effect of schistosity on the long-term stability of large excavations. The comprehensive resolution of these enquiries stands to enhance our knowledge of the performance of layered backfill support systems within the context of stoping mining. Indeed, the previous questions motivated the present study which is focusing on evaluating the strength development of layered backfill and the stress distribution within layered backfilled stope. The approach used in this study integrates laboratory analysis of layered and non-layered backfill performance. This analysis incorporates curing time, strength analysis, deformation analysis, etc. Following that, a 3D numerical simulation was applied to identify the stress–strain distribution of both layered and non-layered backfill. A short discussion on the comparison of the results with the existing literature was included before the conclusions.

## Materials and methods

This section outlines the procedure followed in performing the experimental analysis and numerical simulation. It commences with a detailed description of the ingredients used to prepare the cemented paste backfill and the methodology employed to mould and cure the samples. The numerical simulation procedures are therefore documented in the last part of this section.

### Materials

#### Tailings characteristics

The tailings used in the experiment were collected from old gold mine dumps in the western region of Johannesburg, South Africa. The particle size distribution of the tailings was done as per the recommended standard procedures by ASTM D422^32^.

**Figure 1:**
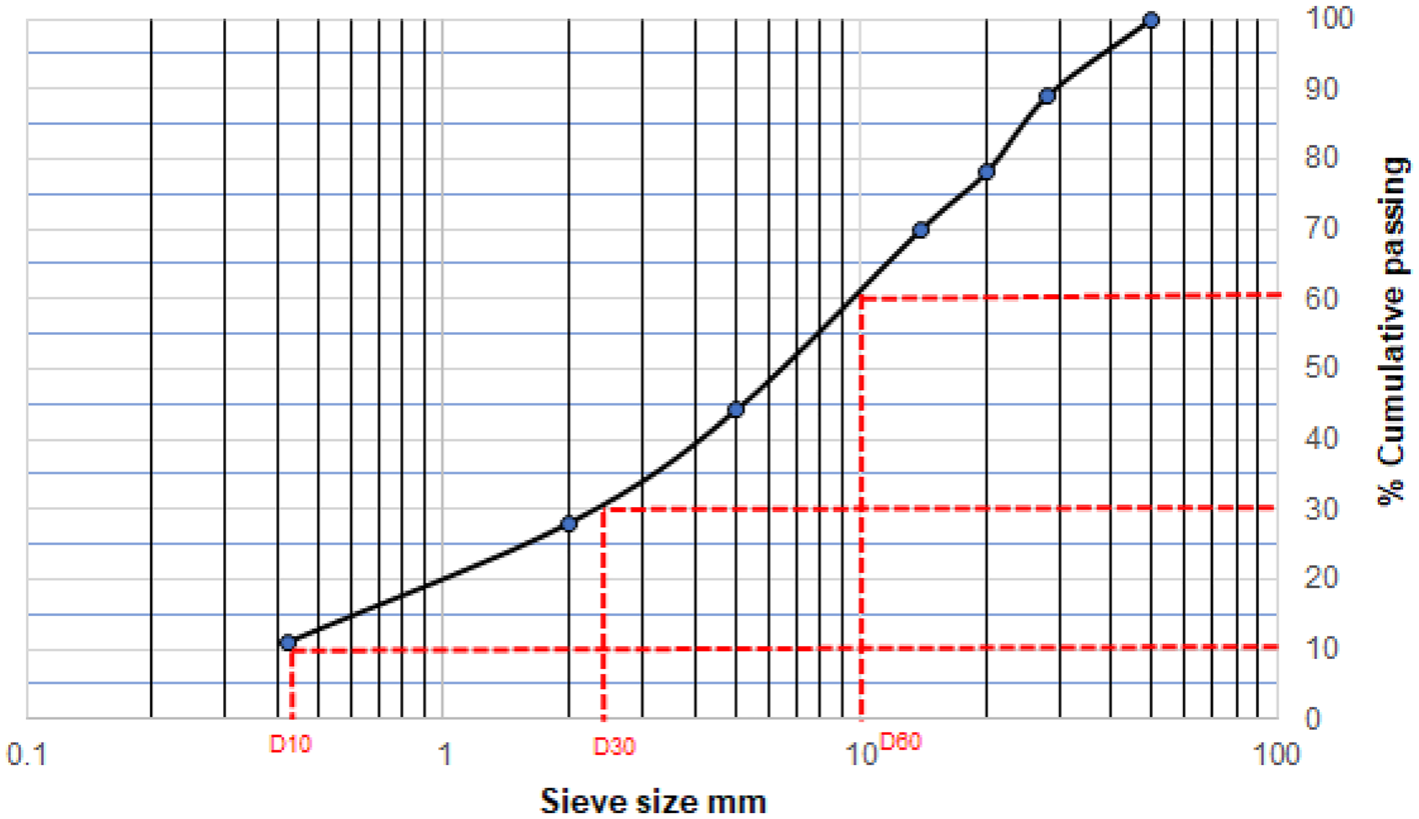
Particle size distribution of the tailings.

The following materials were used: a stack of sieves of different sizes in descending order, i.e. 50 mm, 28 mm, 20 mm, 14 mm, 5 mm, 2 mm, 0,425 mm and < 0.425 mm; a pan at the bottom of the sieves, a plate, weighing scale and a drying oven. The tailings were placed in an oven and dried overnight at 110 °C for 24 h. As depicted in Fig. 1, the 60% D_60_, 30% D_30_ and 10% D_10_ of fine particles that passed through the lowest sieve are 10 mm, 2.3 mm and 0.41 mm, respectively. Therefore, the inhomogeneity coefficient Cu and curvature coefficient Cc are 24.39 and 1.29, respectively. The findings indicate that the tailings are well-graded^33^.

**Figure 2:**
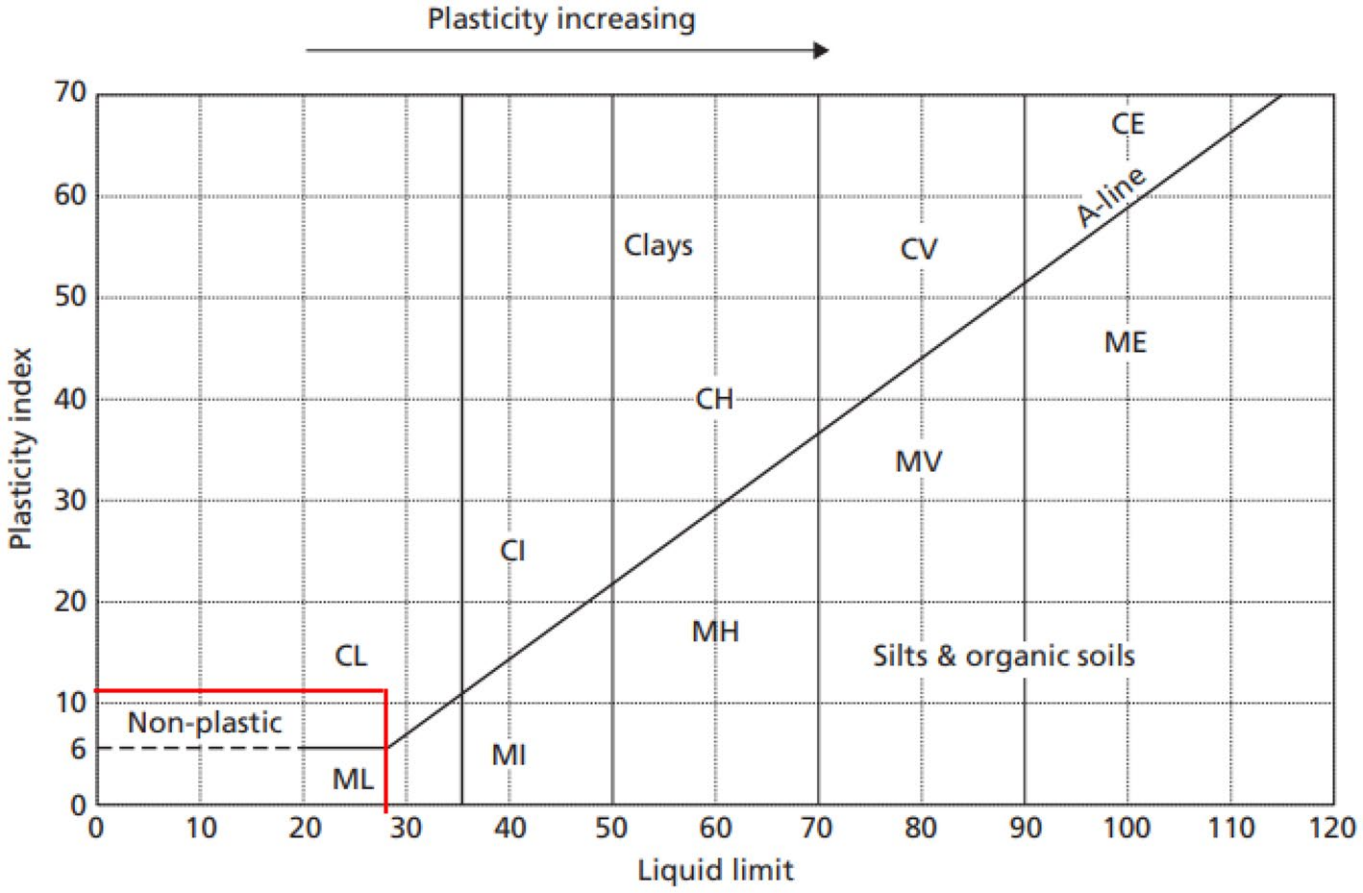
Plasticity index chart to classify the tailings.

Atterberg limits were done to determine the liquid limit (LL) and plastic limit (PL) of the tailings according to the ASTM D4318^34^ standards. The objective of these tests is to classify the characteristics of soil material in terms of its liquidity, plasticity, and solidity. In Fig. 2, it is denoted that the tailings are of a low-plasticity clay material. Indeed, the tailings were sticky and clayey as observed in the lab.

The mineral composition of the tailings was obtained by using the X-ray fluorescence (XRF) technique and the composition is shown in Table 1. The tailings used in this work are very high in sulphide minerals (the tailings dumps have been exposed to air and water for several years, hence, the high pyrite content). Sulphide minerals are known to affect the strength of CTB through a process known as the sulphate attack. The effect of sulphate attack on the strength of CTB in this study was overcome by using CEM V Portland cement rich in furnace slag and fly ash. The cement was particularly manufactured to resist sulphate attack and other harsh conditions.

**Table 1:** Mineral composition of the tailings.

Component	Mass fraction (%)
MnO	0.024
Fe_2_O_3_	3.8
FeS_2_	64.4
Ni	2.4
Zr	2.6
Ru	3.4
Rh	12.8

#### Cement

An Ordinary Portland Cement (CEM V 42.5 N) was used as the hydraulic binding agent. The CEM represents the term cement, whilst the ‘V’ refers to the constituents of the cement. CEM V contains a high percentage of blast furnace slag and fly ash, up to a maximum content of 38%, this type of cement is highly used to resist sulphate attack. The 42.5 N refers to the grade (strength) of the cement and the speed of strength gain, thus 42.5 MPa with normal (N) strength gain. The addition of 20% of fly ash to cement results in reduced water consumption^35^ and the use of slag aids in the reduction of carbon dioxide emissions^36^. However, Krol et al.^37^ add that cement with significant amounts of fly ash and slags generate low hydration heat but obtain higher strength after longer curing periods than traditional ordinary cement.

#### Mix proportion and specimen preparation

**Figure 3:**
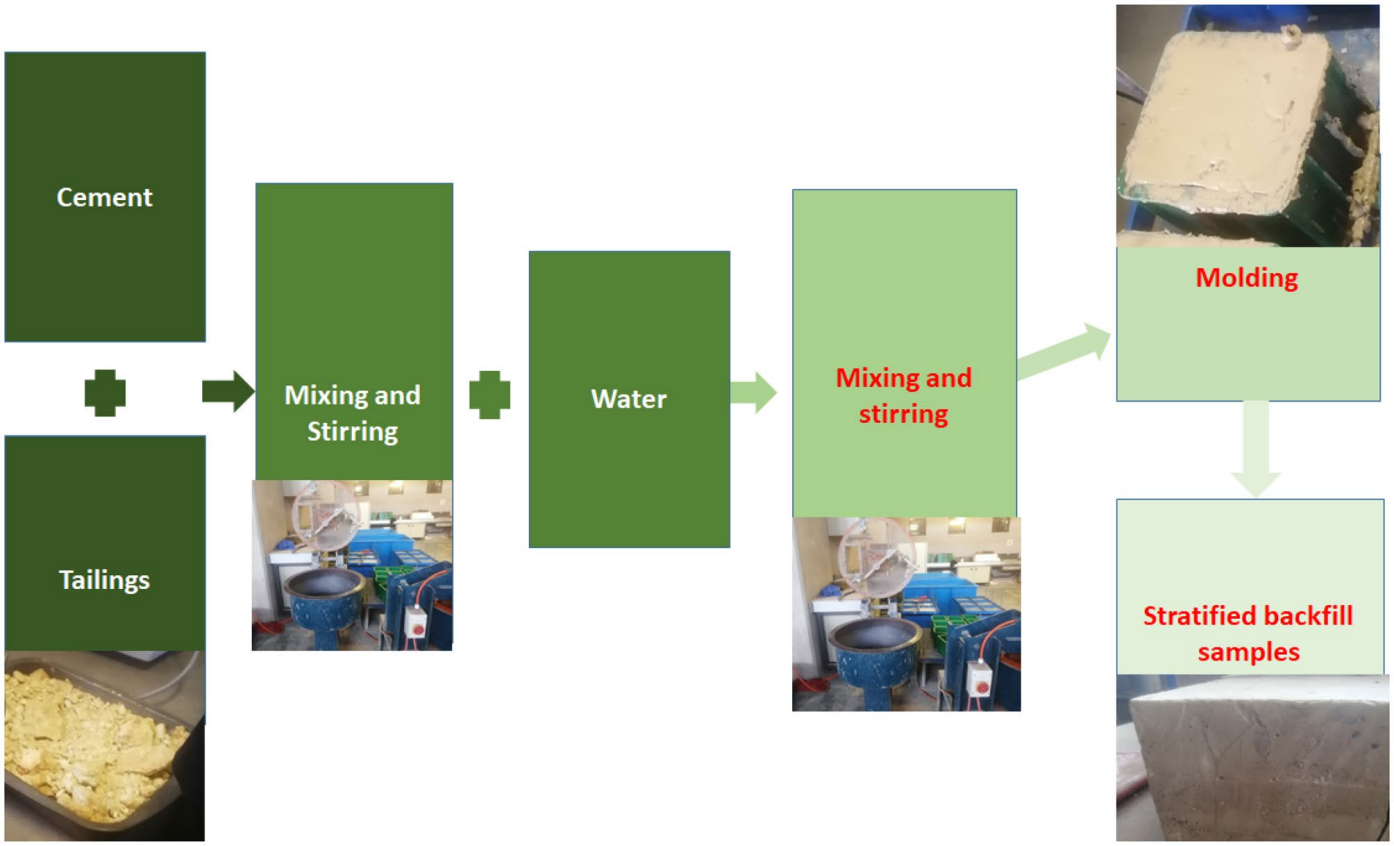
A flow diagram of the procedure followed to prepare and compress the samples.

The CTB specimens were prepared according to the ASTM C 192^38^ standards. The backfill samples were made with a cement/tailing (c/t) ratio of 2.5 which makes up a solid concentration of 73%, and a water content of 27%. The second mixture makes up a cement/tailing ratio of 2.3, which is 10% cement higher than the first mixture to observe the change in the strength of the specimens. The ingredients were mixed using a concrete mixer for at least 10 min, as recommended by Qi et al.^39^, to obtain a homogenous slurry. CTB samples with 1 to 3 layers were prepared using 150 by 150 mm cubes. The non-layered samples were prepared in one day through the one-time filling. The samples with two or more layers were prepared by pouring the first layer and allowing it to cure for 24 h then filling up the following layer. To prevent inconsistencies of the results, the top surface of the preceding layer was scratched to glue the layers together. A total of 108 samples were prepared and the height of each layer was the same. The samples were demoulded after 72 h and cured in water for 7 days, 14 days, 21 days and 28 days after the first filling. The upper layer and lower layer of the samples were smoothed and subjected to UCS testing. Figure 3 illustrates the process explained above.

A slump test was conducted according to ASTM C143^40^ standards, to determine the consistency and transportability of the CTB^41^. The slump values obtained in both mix ratios are 185 mm and 180 mm. According to Belem et al.^42^ and Ouattara et al.^43^ the slump is still within the acceptable slump of 152 and 254 mm. Although the slump value decreased with increasing cement content, the transportability of the paste was not compromised.

### Numerical model setup

A block of 75 by 100 m was developed to simulate the rock mass located underground. The block was discretized into 10 000 elements with the consideration that a mesh size that is too coarse may generate inaccurate results. A block size of 30 × 25 × 20 m was excavated in the rock mass to simulate a 20 m open stope. The properties of the rock mass and backfill are adapted from literature and are shown in Tables 2 and 3. The rock mass is assumed to be homogeneous, and isotropic and obeys the Mohr–Coulomb (MC) properties. The results from the Atterberg limit indicated that the tailings are clayey. Therefore, the backfill material was also assumed to be stiff clay and obeys elastic–plastic behaviour^44^.

**Table 2:** Properties of the rock mass used in the numerical modelling (Guo et al.^45^, Qi et al.^46^, and Zhao et al.^47^).

Parameter	Value
Bulk density (kg/m^3^)	2700
C (MPa)	5.5
Shear modulus G (MPa)	12 200
Bulk modulus K (MPa)	26 400
E (MPa)	0.66
Unit weight	28
Ø friction angle (°)	57
Poisson ratio	0.3

**Table 3:** Properties of the backfill material used in the numerical modelling (Guo et al.^45^, Qi et al.^46^, Zhao et al.^47^).

Parameter	Value
Bulk density (kg/m^3^)	2700
C (MPa)	20
Shear modulus G (MPa)	62.5
Bulk modulus K (MPa)	83 300
E (MPa)	150
Unit weight	20
Ø friction angle (°)	30
Poisson ratio	0.3

The input material properties used for the model is adapted from Guo et al.,^45^, Qi, et al.^46^, and Zhao, et al.^47^, as shown in Tables 2 and 3.

#### Initial stresses

The initial stress σ_0_ was calculated using the overburden height of 1000 m, the density of the material of 2700 kg/m^3^ and the gravitational constant. Equation (1) was used to compute the initial stress and was found to be 26.5 MPa. The pressure coefficient (K_0_) is assumed to be 0.5.

1 $$ \sigma_{0} = \, \rho .h.g $$

#### Backfilling process

**Figure 4:**
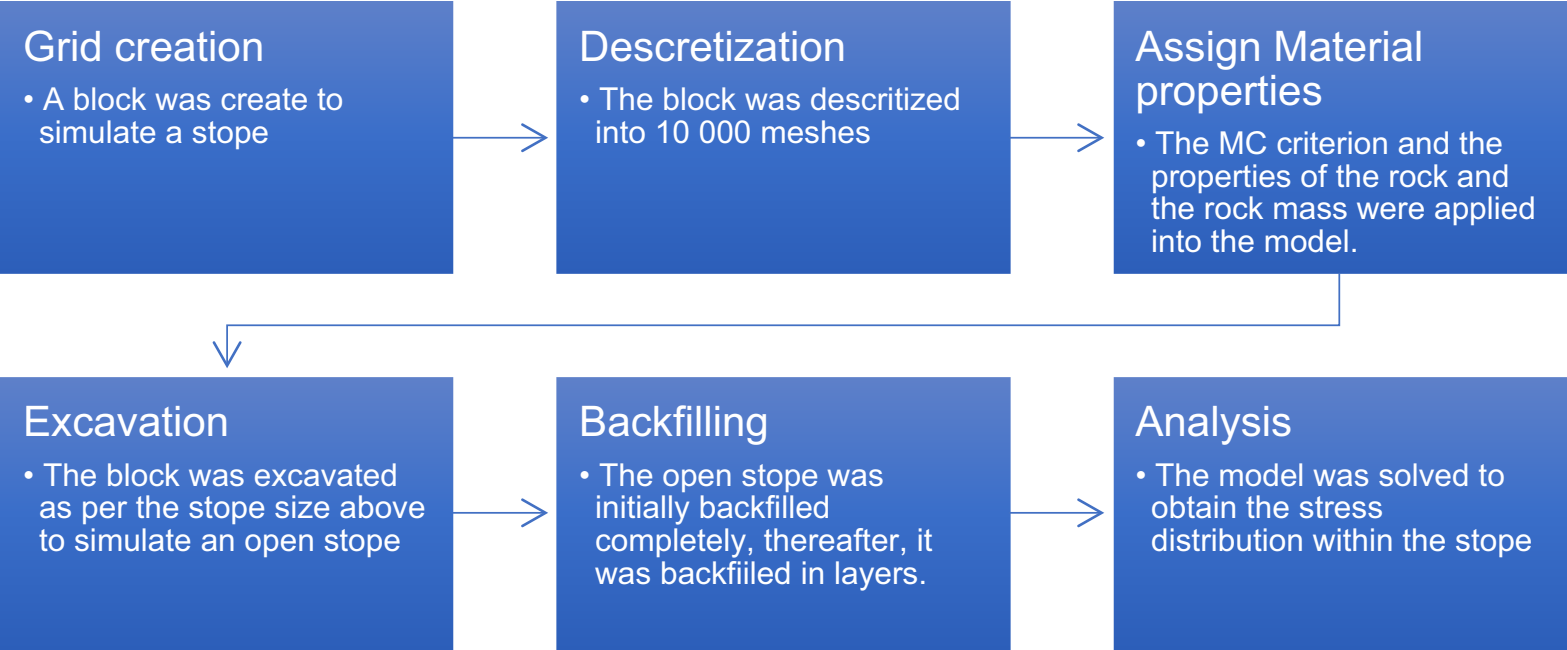
Illustration of the backfill modelling process.

The 20 m stope was backfilled using different strategies, i.e. an immediate single pour, two layers of backfill and consecutively to four layers of backfill. It is assumed that the stope is backfilled from the top, thus, a tight fill is achieved. Therefore, no gap is left at the top of the stope. The simulation of each filling strategy is run from the excavation stage until backfilling is complete. After the excavation, the backfilling process is simulated as the material is placed in layers until the stope has been backfilled. The schematic view of the modelling procedure is shown in Fig. 4.

## Results and discussions

### Effect of curing period on the strength development of CTB

**Figure 5:**
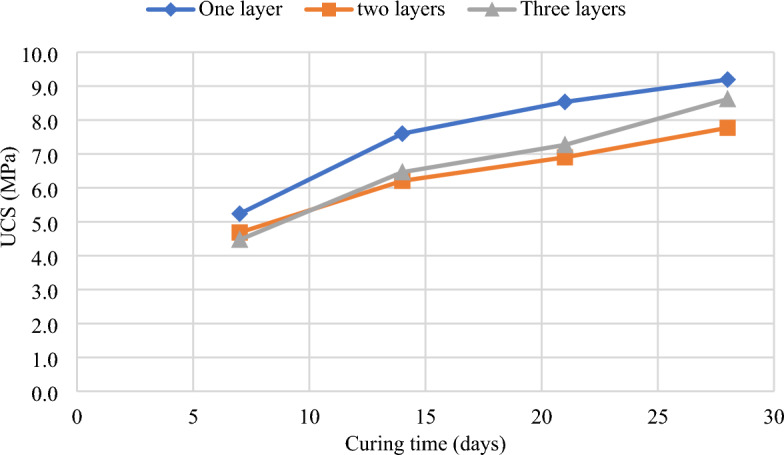
The effect of the curing period and cement content on the UCS of CTB samples^50^.

Figure 5 illustrates the effect of the curing period on the UCS development of CTB samples. The UCS of the samples develops with the curing period regardless of the binder dosage or type. For example, the percentage increase of UCS from 7 to 14 days for non-layered samples is 31.08%, whereas the percentage increase after 14 and 28 days is 10.97% and 7.16%, respectively. Meanwhile, the rate of increase for two-layered samples from 7 to 14 days and 14 to 28 days is 24.64%, 10%, and 11.2%. In this case, we see a high increase rate of UCS at an early age, then a slight decrease, and thereafter it climbs again. A similar trend is observed for three-layered samples at 30.80%, 11.14% and 15.66%. However, although the rate of increase for layered CTB is uneven, the UCS is still increasing with curing time. The findings show that non-layered backfill samples gain more strength with time than layered backfill samples.. The strength development is high at an early age (7–14 days) as compared to long-term strength development (21–28 days). The strength gain is due to increased cement hydration products such as C–S–H gels, which are known to reduce porosity and improve the cohesion of CTB^48^. The formation of hydration products is high at an early age, thus, contributing to the high growth of UCS^49^.

### Effect of layering on the UCS of CTB

**Figure 6:**
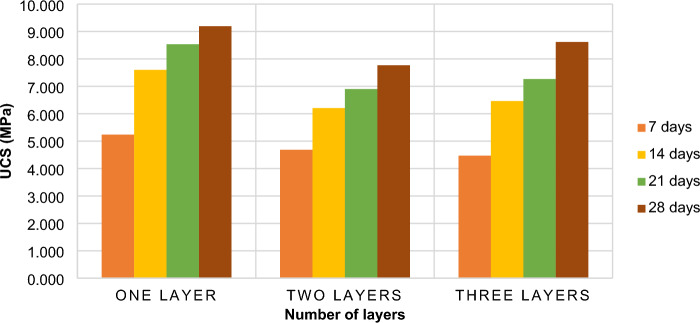
The effect of layering on the UCS of CTB samples.

Figure 6 depicts that the strength of non-layered (complete filling) samples gained more strength than two-layered and three-layered samples. The strength of the samples decreases with increasing layers. Although a slight increase in strength is observed in three-layered samples. This trend of strength loss is due to the backfilling gap adopted as practised in the mining industry since the bottom layer is poured first, the 24-h curing period allows the bottom layer to gain some strength. Following that, for layered samples; the second layer, and so on are expected to have less strength than the bottom layer. Likewise, the variation in strength between the layers (first, second and third) is expected. In this study, curing periods of 7, 14, 21 and 28 days were implemented, therefore, the differences were noticeable. Other authors that have tested layered CTB samples cured their samples for more than 60 days to minimise the effects of time differences^14,27,50^. These authors reported an exponential decrease in the UCS of CTB samples with increasing layers.. Indeed, as much as the UCS of the samples decrease with increasing layers, when the number of layers were 3 the UCS started to increase. Therefore, it can be concluded that as much as the non-layered CTB structure possesses more strength than the layered CTB, the strength of layered CTB samples is not always dwindling. By fitting the data, it is evident that the quadratic polynomial function is more consistent with the given data. Wang et al.^14^ also found that the quadratic polynomial function was the best fit to describe the behaviour of the UCS of layered CTB samples with increasing layers and the authors also used gold mine tailings. This provides a positive correlation with the current results.

### Effect of layering on the elastic modulus of CTB

**Figure 7:**
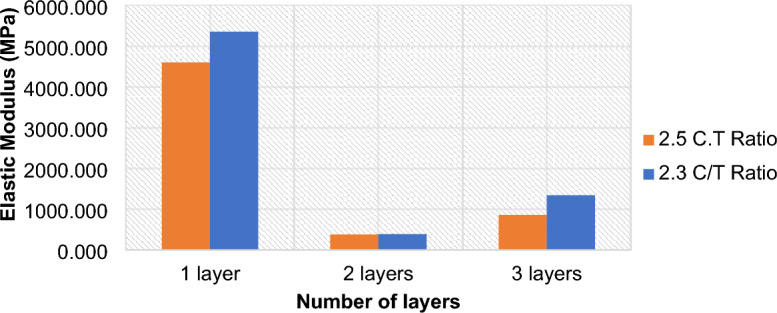
Effect of layering on the elastic modulus of CTB samples.

The elastic modulus, as shown in Fig. 7, of non-layered samples, two-layered samples and three-layered samples is 4596.7 MPa, 381.8 MPa, and 861.6 MPa after 28 curing days. The findings show that the non-layered samples are stiffer than the layered samples, thus, it will require high stress to deform non-layered samples than is required to damage layered samples. The deformation of layered CTB samples occurs due to the weaker upper layers that are cured later than the bottom layer. As observed during the compressive tests, the top layer suffered the highest damage while the second layer would absorb some of the energy from the upper layer. Consequently, the second layer will be damaged too but at a lower scale. Therefore, there would be some residual strength endured by the bottom layer, resulting in a loading and unloading stress/strain behaviour of the samples.

The elastic modulus of the 2.1 c.t mix ratio is also higher due to the effect of cement content. Indeed, the higher the cement dosage, the stiffer the sample. Therefore, an increase in cement dosage would be another way to improve the strength of backfilled stope in an underground environment. However, this would also result in higher cement consumption, which accounts for more than 60% of mine’s backfilling cost.. These findings further confirm that the strength of layered CTB does not always decrease with increasing layers. This pattern was observed in the laboratory during the compression tests. The samples with two-layers were easily dislodged and the upper layer was the one incurring more damage than the lower layer. Whereas the three-layered samples would still remain as a single layered solid sample, moreover, the deformation of the sample would be in a form of a crack propagating from the top to the bottom layer. As a result, the three-layered samples recorded a higher UCS than the two-layered samples, and consequently, a higher elastic modulus than two-layered samples.

### Failure patterns

**Figure 8:**
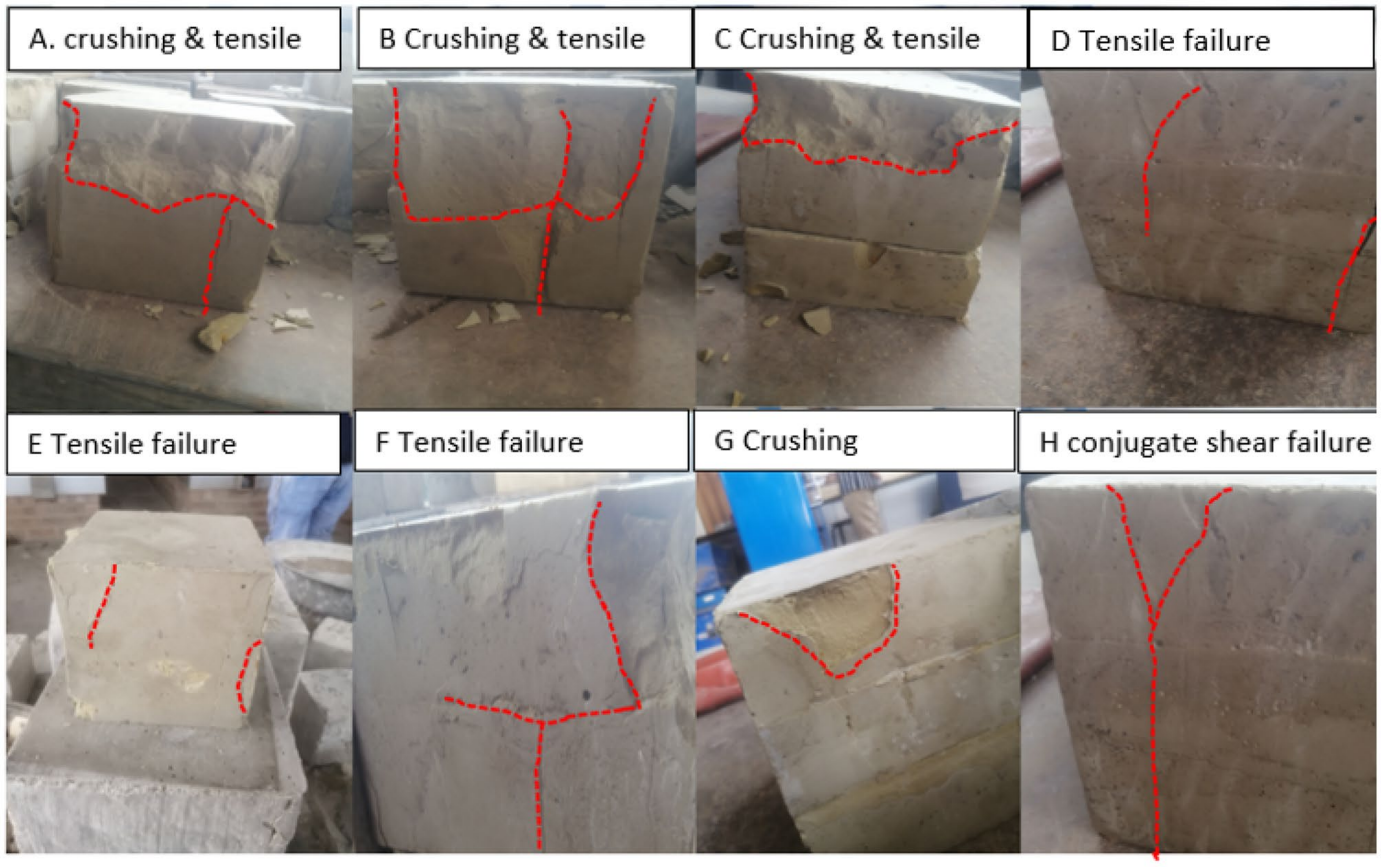
Failure patterns of backfill specimens with different layers.

The failure characteristics of layered and non-layered backfill samples are depicted by Fig. 8. A camera was used to capture common failure patterns demonstrated by the samples. The failure mode of non-layered CTB samples is mainly characterized by tensile cracks^51^, this failure pattern is common in all curing ages for non-layered backfill samples. Whereas two-layered CTB samples are characterized by surface spalling of the upper layer (loading area), also known as crushing^52^. The crushing of the loading area was severe at 7 days, however, the damage of the loading area decreased with more curing days (since the hardening of the samples increased with curing time). Moreover, the bottom layer of the two-layered samples was less deformed during the compression of the samples in all curing days. This was due to the displacement of the two layers from where they were joined, thus, only resulting in the deformation of the upper layer. The failure mode of three layered CTB is characterized by a main shear crack and a tensile crack, which intersect to form a Y-shaped shear-tensile or conjugate shear failure^53^. Three-layered samples were more glued together than two-layered samples, hence, the three-layered samples only endured a crack from the top layer to the bottom layer. However, the samples were never disjoined together. Consequently, this results in more strength than two-layered samples. This phenomenon explains the increase in the strength and elastic modulus of the samples as the layers increase.

### Numerical modelling

This section presents and discusses the results of stress distribution in a backfilled stope from the numerical analysis. The first backfilling strategy to be analysed is the single pour, followed by the double-layered fill to the four-layered backfill. The results show the compressive principal stress, strain, and shear dissipation within the stope.

#### Case 1: open stope

**Figure 9:**
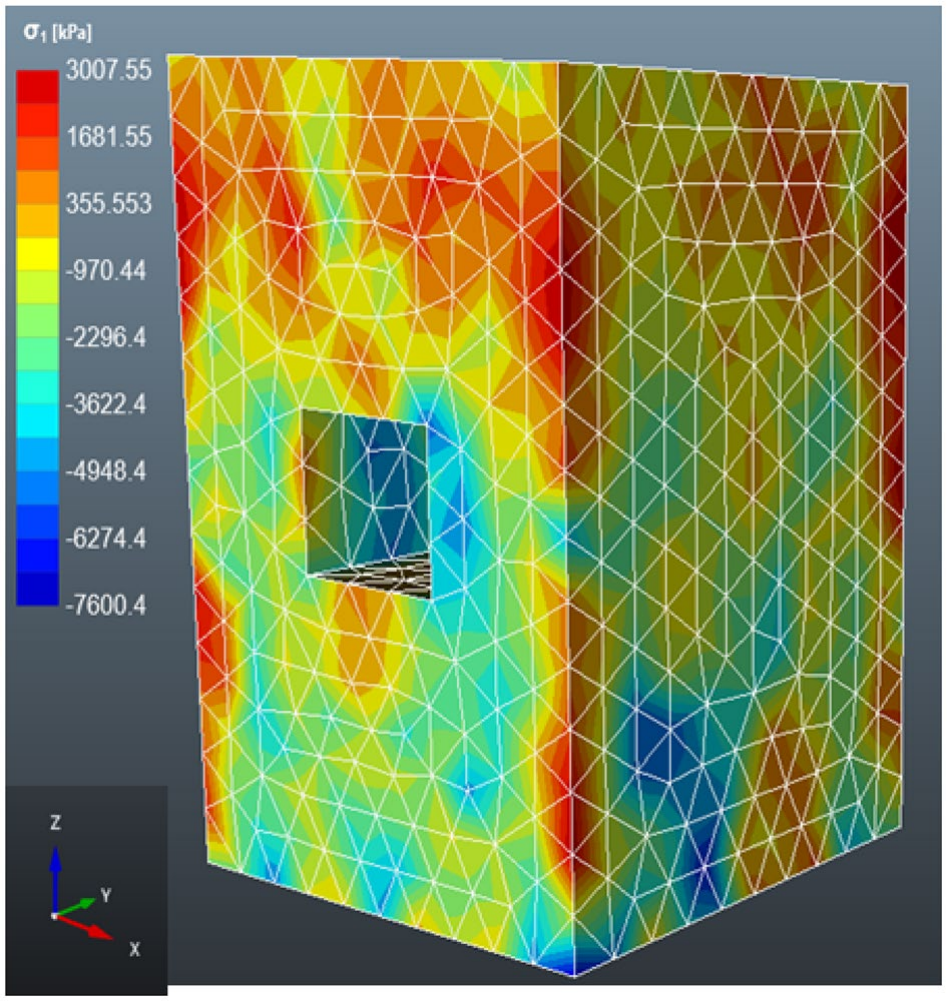
The distribution of the major principal stress (σ1) in the open stope.

After the extraction of the ore in the stope, the numerical analysis shows that the principal stress is higher at the outer boundaries of the rock mass (see Fig. 9). Whereas the principal stress is low at the boundaries of the excavation. Moreover, the principal stress is high at the rock mass at the hanging wall, this may be due to the stope closure triggered by the excavating/extraction activity^54^. The stress distribution around the opening is distributed to accommodate the disturbance in the stress field^55^.

#### Case 2: non-layered backfilled stope

**Figure 10:**
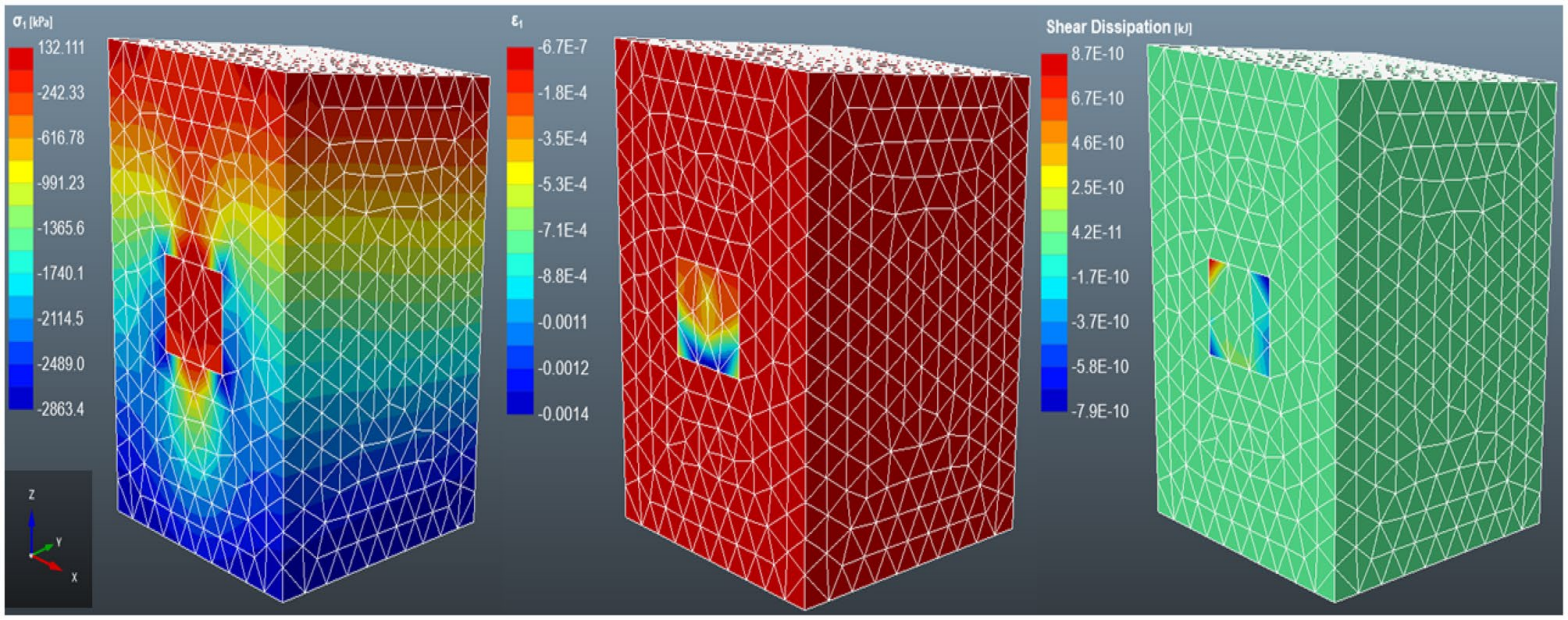
The distribution of the major principal stress (σ1), major strain stress (ε1) and shear dissipation in a non-layered backfilled stope.

Figure 10 depicts the stress transfer from the rock mass to the backfill material. The principal stress is high at the top of the rock mass and the backfill material. Meanwhile, a rapid stress decrease is observed continuously towards the bottom of the rock mass. The stress level is completely low at the bottom of the rock mass, signifying the presence of stress transfer between the backfill and the rock mass^55^. Therefore, failure will be expected at the backfill pillar. Qi et al.^46^ who performed a numerical analysis of layered backfill and this study also adopted the same material properties as their study, postulate that tight backfilling reduces the degree of displacement of the rock mass. The rock mass located at the bottom of the backfill is also experiencing some stress as it is also carrying the load from the settlement of the backfill. However, the major principal stress has declined from 3 to 0.132 MPa (95.6% decrease). The findings show a high strain on the top portion of the backfill support with minor horizontal strain on the sidewalls of the backfill. This may be an indication of confinement to the backfill from the sidewalls due to the horizontal stress^56^. The deformation of the fill tends to intensify in the rock mass and the upper part of the backfill. Whilst the bottom part of the fill is the least deformed, thus, the change in the volume of material would be expected on the upper part of the backfill. Moreover, there is evident shear dissipation along the interface between the backfill and the rock mass, as well as the backfill interfaces. The shearing between the interfaces is due to cohesion, interface roughness and interface friction angle at the interfaces^57^. The negative stress and strain within the rockmass and the stope are due to compressive stress whilst the positive stress and strain signify tensile stress. Therefore, as shown in Fig. 10, the bottom part of the backfill is highly compressed as compared to the other areas of the backfill.

#### Case 3: double layered backfilling

**Figure 11:**
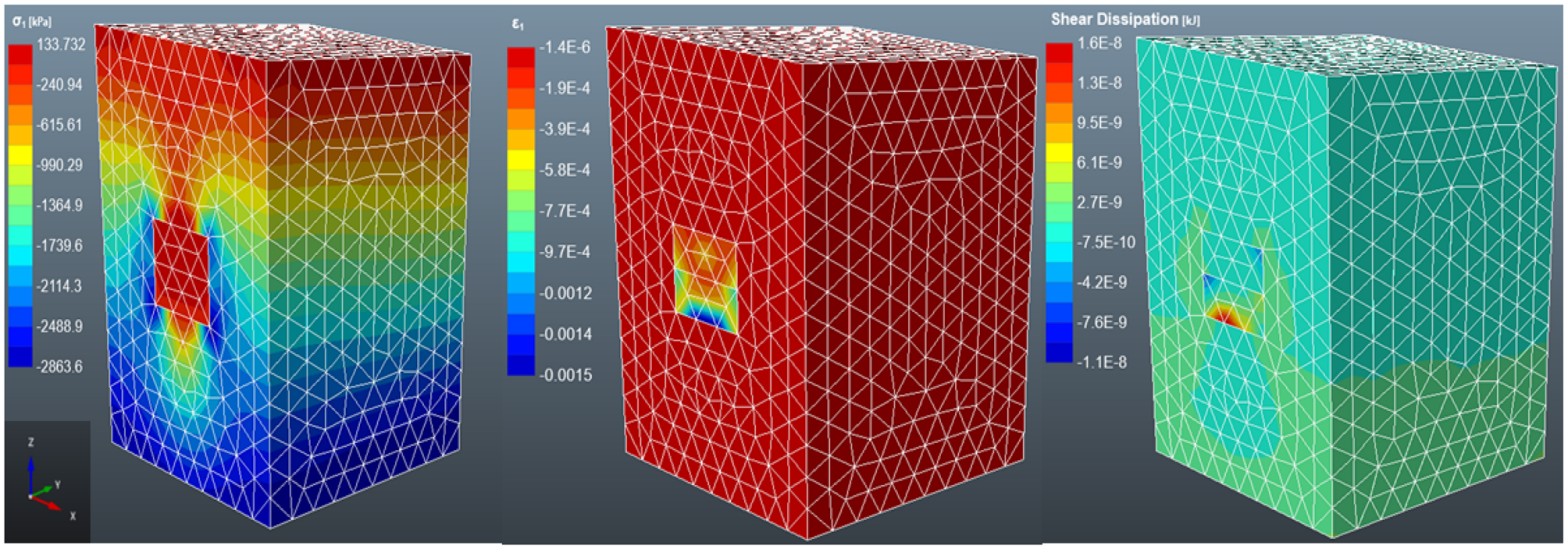
The distribution of the major principal stress (σ_1_), major strain stress (ε_1_) and shear dissipation in a two-layered backfilled stope.

The stress distribution in a two-layered backfill is similar to single-layered backfill (see Fig. 11). However, the major principal stress has declined from 3 to 0.134 MPa, which is only a 1.51% difference from the single-layered backfill. Therefore, there is only a slight decrease in the strength of the backfill from non-layered to two layers. The strain also expands from the top layer to the bottom layer. However, the strain in the bottom layer is not as high as the strain in the upper layer. Meanwhile, for non-layered backfill, the shear dissipation was along the rock mass-backfill side interfaces, whereas in two-layered backfill, the shear dissipation is visible at the bottom rock-fill interface and along the backfill layers interface. This shows that there is some shearing also occurring between the layers. The settlement of the backfill generates shear stress at the rock-fill interface, the shear stress is determined by the level of friction at the interface^57^. There are no documented findings to support the characterisation of the shear dissipation along the backfill layers.

#### Case 4: three-layered backfilling

**Figure 12:**
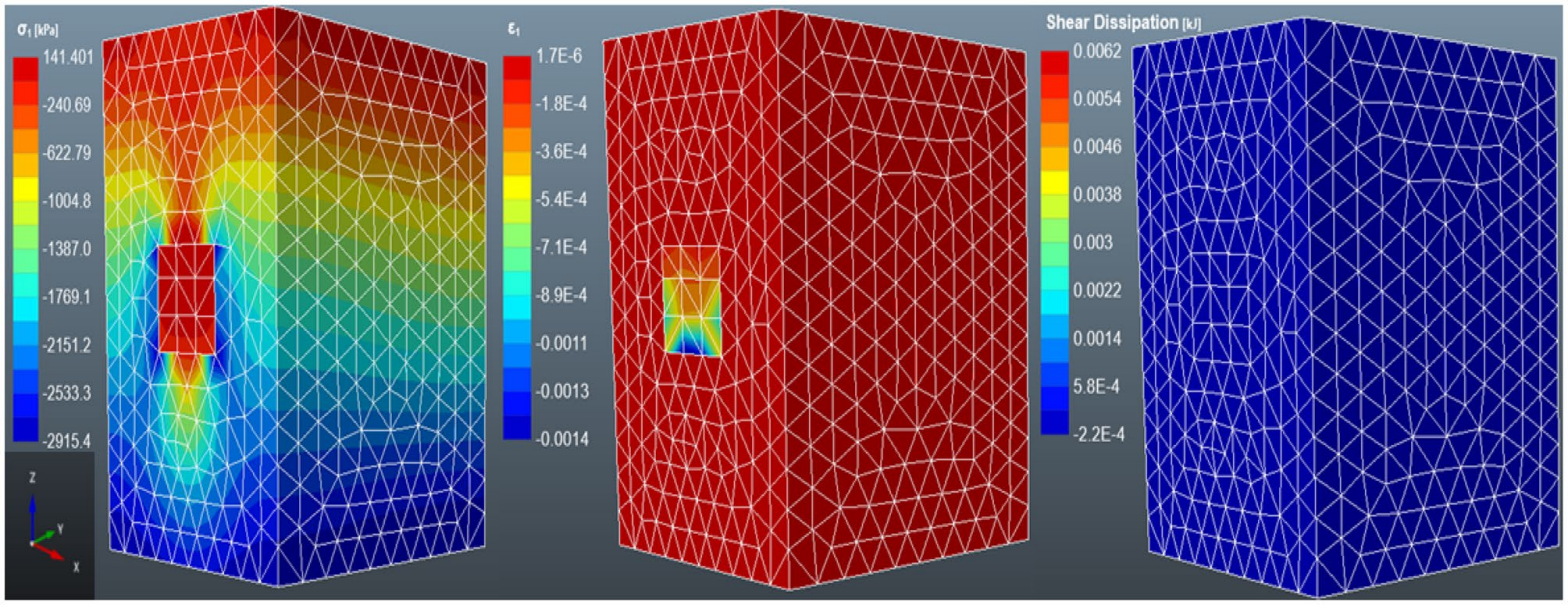
The distribution of the major principal stress (σ_1_), major strain stress (ε_1_) and shear dissipation in a three-layered backfilled stope.

According to Fig. 12, the major principal stress and strain increase exponentially with an increase in layering. The major principal stress after the filling is 0.141 MPa, i.e. a 6.38% and 4.96% increase from the non-layered and two-layered backfill. While the stress distribution is the same as the non-layered and two-layered backfilled stope, there is evidence of high shear dissipation in the three-layered backfill as compared to the other cases. The cause of this is unknown, however, it is worth noting that the layers for the three-layered backfilling strategy are not equal in length, unlike in other cases. The stope was filled with two 7 m layers plus one 6 m layer to a reach 20 m stope. Similarly, Chen et al.^31^ investigated the mechanical properties of layered backfill. The group of researchers observed a decline in the strength of the samples as the number of layers increased. Moreover, Chen et al.^31^ did not conduct a numerical analysis of layered backfill, thus, these findings are based on laboratory work.

#### Case 5: four-layered backfilling

**Figure 13:**
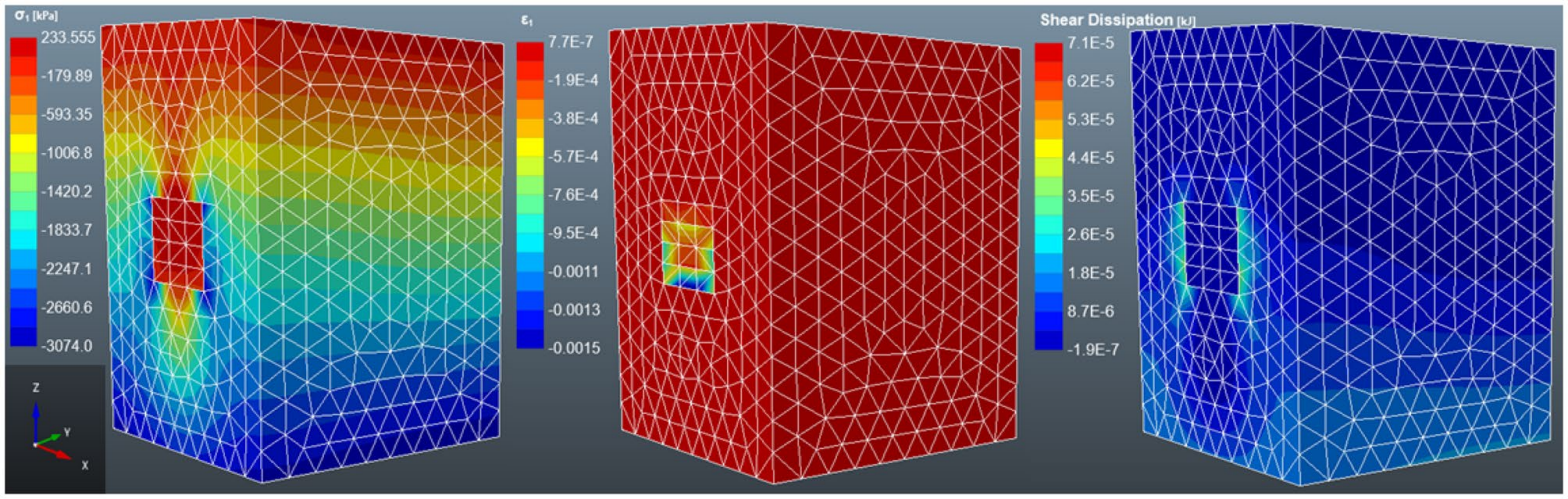
The distribution of the major principal stress (σ_1_), major strain stress (ε_1_) and shear dissipation in a four-layered backfilled stope.

In the case of four-layered backfill (see Fig. 13), the major principal stress, major strain and shear dissipation have increased significantly compared to non-layered backfill. The principal stress and strain have increased by 77.27% and 14.93%, respectively. Whilst the shear dissipation has spread to the lower boundaries of the rock mass. The strain also intensifies towards the inner parts of the backfill, but it does not affect the bottom layer of the fill body. These results are supported by Chen et al.^58^, the authors used a micro-mechanism technique to study the damage of layered backfill samples. They report that as the number of layers increases, there is excessive accumulation of damage in the middle layer. Therefore, the load is not transferred to the bottom of the backfill body and thus, maintains a certain strength. Qi et al.^46^ attest that backfilling in layers affects the plastic deformation of the upper and middle layers of the backfill but has a minor impact on the stress distribution of the lower part.

### Deformation curves

**Figure 14:**
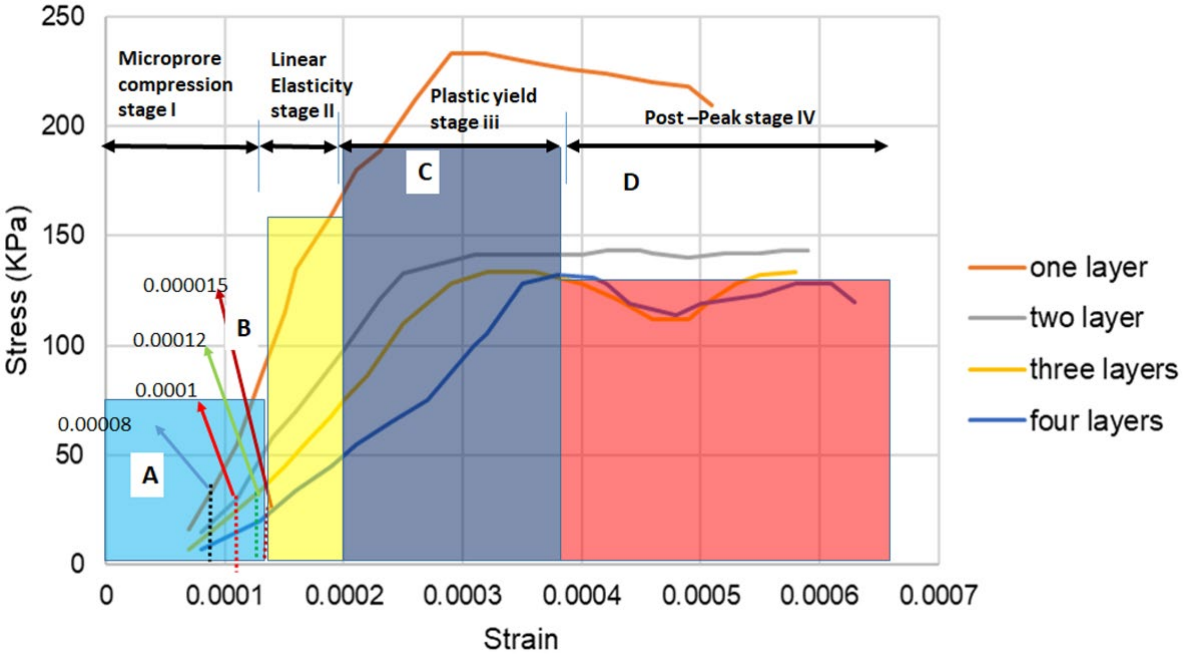
Stress–strain curve for four-layered backfill.

The stress–strain curves in Fig. 14 were generated from the numerical modelling by analysing the change in strain as the stress scale increases. The stress–strain behaviour of the backfill is consistent with the observations made by several authors^55,59,60^. The deformation of the CTB can be divided into four stages (see Fig. 14):

- Micro-pore compaction stage (A): at the beginning of the curve, a concave shape is observed, which indicates the compaction of the internal pores of the CTB structure under compressive pressure. It is at this stage that the curve becomes a straight line, and the deformation enters the linear elastic stage.
- Linear elastic stage (B): this stage is denoted by the strain line curve. This is where the internal pores of the CTB are further compacted as the confining pressure continues to increase. However, the pressure does not cause any cracks in the CTB, and this stage is representing the elastic modulus of the material.
- Plastic yield stage (C): In this stage, cracks are generated when the stress reaches its highest peak. The inner cracks expand gradually and worsen until the rapture. As a result, the curve exhibits a convex upward shape.
- Post-peak stage (D): The microcracks continue to expand and gradually evolve into primarily visible cracks. Thus, as the cracks propagate, the load-bearing capacity of the CTB decreases. Consequently, the CTB structure has become fully damaged.

According to Fig. 14, the deformation behaviour of nonlayered CTB shifts from ductile to brittle behaviour (CD). Similar findings are recorded by Liu and Fall^55^. This phenomenon is known as the strain hardening/softening behaviour^44,61^. The authors attribute this deformation behaviour to the increased stiffness of the CTB structure. The increase in confining pressure results in a change in the mode of failure of the CTB at peak stress^44,61^. The elastic modulus of the backfill was measured by calculating the gradient of slope AB.

The deformation curve of the two-layered backfill does not differ much from the non-layered backfill. Even the stress in the backfilled stope increased by 1.51% from non-layered to two-layered backfill. As shown in Fig. 14, the last stage displays a minor fluctuation in the deformation curve. This may be caused by the undamaged second layer of the backfill. When the first layer has failed, the second layer remains intact as observed during the laboratory experiments^13^. Similar findings were observed by Wang et al.^14^ in their experimental investigation of the mechanical behaviour and damage evolution of layered backfill. According to the group of authors, when the load exceeds the maximum pressure-bearing capacity of the sample, the sample becomes unstable and deformed. However, due to the residual strength of the sample, the stress–strain curve does not decrease rapidly but advances slowly forward. The elastic modulus for the two-layered backfill is lower than the non-layered fill.

On the other hand, the three-layered backfill perfectly obeys the elastoplastic deformation behaviour. The deformation behaviour of three-layered backfill goes through four different stages as discussed previously. Initially, the interface of the different layers in the CTB remains intact under the continuous load and the internal cracks are continuously compacted. This stage is denoted by the concave shape at the beginning of the stress–strain graph. Furthermore, the layered backfill gradually exhibits elastic properties as the pores are further compacted. The linear growth in the graph demonstrates this behaviour. When the material has reached the peak strength, the stress–strain curve shows a slow decrease trend after the peak. Thus, the CTB material indicates a ductile failure pattern^50^. Likewise, the elastic modulus has decreased with increasing layers.

The deformation curve of the four-layered backfilled depicts loading and unloading failure characteristics^14,62^. The post-peak point (CD) depicts a zigzagged shape, i.e. the peak stress experiences the rise and fall pattern. The initial stress increase is due to the instability of the top layer then the stress reduces due to the elastic deformation of the next layer. Eventually, when the next layer goes through the crack propagation stage, the stress rises again, and the process repeats itself. Zhang et al.^63^ reported similar deformation behaviour. However, the authors investigated the deformation behaviour of CTB in a sub-zero temperature environment. The UCS rise and fall were attributed to the hydration process at an early age and the effect of freeze–thaw, respectively. This deformation cycle may eventually lead to the deterioration of the CTB structure. The four-layered backfill has the lowest elastic modulus. The elastic modulus for the backfill with increasing layers is 1 × 10^06 MPa. 714,910 MPa, 511,055 MPa, and 375,026 MPa.

Further analysis was to look into deformation stages presented by the model with regards to the CTB material within the stope. It was denoted that the deformation has shown four deformation behavior of the CTB, these results correlate very well with some of the recent studies such as those of Chen et al.,^55^. Following that, Chen et al.,^31^ four deformation stages were presented, and those stages include “(I) a micro-pore compression stage, (II) a linear elastic stage, (III) a plastic yield stage, and (IV) a post-peak stage” similar results were observed as denoted by Fig. 14. Indeed, one may point out that the no layered backfill has demonstrated very low deformation in stage I as compared to the various layered backfill support system. A similar trend has been observed by Chen et al.,^58^ and others as documented in the previous sections. It is believed that the introduction of layered CTB has contributed largely to the micro-pore compression stage of the stress–strain curve which changes to the micro-pore and this micro-pore initiates crack quicker as compared to the non-layered backfill. One may also support this argument by looking at some of the theories that govern the development of fractures as stated by Sengani^64^, yet similar principles appear to be evidence in this case. Similarly, the deformation, in this case, corresponds to less stress-induced backfill support system in layered as compared to non-layered backfill. This solidifies the argument documented above that a non-layered backfill gains more strength than a layered backfill system while it presents less deformation with time. One may also denote that the effect of shearing may also have played some cardinal role in the deformation process of stratified backfill.

In stage II the simulation validated the laboratory results as well. In this regard, the non-stratified backfill appeared to present a high elastic modulus yet the stratified backfill system appeared to experience a low elastic modulus. Indeed, one may point out that elastic modulus is directly proportional to the stress, yet the increase in stress required to deform non-layered backfill is always higher than the stratified backfill support system. Looking into elastic deformation the stratified layered backfill support system presents large deformation as compared to the non-layered, these results correlated very well with studies such as Chen et al.^58^, Li et al.^65^ and Gao et al.^66^. These results provide confidence that a non-layered backfill system should be expected to perform better as compared to a stratified backfill system, therefore at this stage, one may argue that most stopes which have resulted in extensive dilution or collapse could have been those supported with stratified backfill support system yet understand on what could have contributed to such failure was not well established. Following that, a significant observation has been made and it was deduced that the increase in stratified layers has an impact on elastic deformation the increase in layering also contributes largely to the elastic deformation of the backfill support system. Scientifically, it has been argued by various authors such as Chen et al.^31^, and Gao et al.^66^ that even though backfill support systems cure with time but the stratified backfill does not necessarily cure with similar duration as non-stratified, this leads into micro-pore remaining open while crack propagation occurs quicker when tested (see stage III in Fig. 14). However, the understanding of how duration affects the performance of the two backfill systems is not well established, yet it could be considered for future research. Lastly, as the stress applied to increase both backfill support systems stabilized (see stage IV in Fig. 14) however, the elastic deformation follows a similar trend, though it is noteworthy to document that the elastic deformation occurs gradually after the material has reached the so-called plastic yield to post-peak. Though a tall claim cannot be made at this stage a better insight into the performance of backfill (stratified and non-stratified) can be deduced but there are other aspects of the study such as how temperature, crack propagation and duration of curing affect the performance of the two backfill support system has to be well established.

## Conclusion and outlook

In this paper, the strength development of layered backfill is studied with curing time and changing the number of layers. The main findings are summarised below::

1. The strength gain of non-layered CTB samples is much higher than that of layered samples. Furthermore, the strength of layered samples increases with curing time regardless of the layering. Although the strength of the layered samples decreases with increasing layers, the strength does not always decrease but eventually picks up. A similar trend is observed concerning the elastic modulus of the layered samples with increasing layers.
2. The strength evolution of this study was monitored through different curing periods, i.e. 7,14,21 and 28 days. While the findings of the other notable authors who studied the mechanical properties of layered backfill only looked at one curing period, i.e. after 60 days to minimise the impact of curing time. Since this study focused on the strength development of layered CTB, the effect of curing time on the performance of CTB samples could not be ignored in order to assess the change in CTB’s strength with time.It is evident that as curing time increases, non-layered backfill samples gained more strength than layered backfill samples.
3. The numerical analysis reveals that the stress within the stope increases with increasing layers of backfill. Moreover, the elastic modulus of the backfill decreases with increasing layers. When it comes to shear dissipation, there is evidence of shearing along the backfill-rock mass interface and backfill layers interfaces. Thus, indicating the effect of layering on the stability of the fill. Another worth mentioning finding is that the deformation of the backfill seemed occur at the top and middle layers of the backfill while the stress distribution within the bottom layer is unaffected.

Thus. the results show that backfilling in layers reduces the stability of backfilled stopes.

This study, therefore, recommends that further research work be done to investigate the effect of other several factors such as porosity, water content, temperature, and tailings properties, amongst others. This is to ensure that realistic stability charts are developed to predict the stability of exposed backfilled stopes when mining the adjacent stope. The development of predictive charts to predict the next blast is also recommended. Another experimental study to compare the UCS of layered CTB samples of the same binder content with layered samples of different binder content is recommended. This study may lead to the reduction of binder cost for mines using layered backfill support. The study of the strength development of layered backfill is prominent for the mining industry so that the production cycle can be reduced and improve both production and safety.

## Data Availability

The datasets used and/or analysed during the current study available from the corresponding author on reasonable request.
